# Possible de novo clear cell carcinoma in the contralateral ovary 9 years after fertility-sparing surgery for Stage IA clear cell ovarian carcinoma

**DOI:** 10.1007/s13691-016-0271-9

**Published:** 2016-11-24

**Authors:** K. Nishida, Y. Tenjimbayashi, N. Tasaka, A. Shikama, M. Sakuraiv, S. Nakao, H. Ochi, T. Minaguchi, T. Satoh

**Affiliations:** Department of Obstetrics and Gynecology, Faculty of Medicine, University of Tsukuba, 1-1-1 Tennoudai, Tsukuba, Ibaraki 305-8575 Japan

**Keywords:** Early stage epithelial ovarian cancer, Fertility-sparing surgery, Clear cell carcinoma, Clear cell adenofibroma, Relapse

## Abstract

A patient who underwent fertility-sparing surgery for Stage IA clear cell carcinoma may have developed de novo clear cell carcinoma in the contralateral ovary 9 years later. She underwent fertility-sparing surgery and postoperative adjuvant chemotherapy for right ovarian carcinoma at 33 years of age (when endometriosis was observed in the contralateral ovary). At the age of 41 years, a tumor was discovered in the left ovary. This was diagnosed pathologically as clear cell carcinoma with clear cell adenofibroma, which may have developed de novo. A consensus is currently taking shape that although fertility-sparing surgery is a therapeutic option for patients with Stage IA clear cell carcinoma, long-term outpatient monitoring is advised to watch for its recurrence or de novo development in the contralateral ovary.

## Introduction

We encountered a patient who had previously undergone fertility-sparing surgery (FSS) for Stage IA clear cell carcinoma (CCC) 9 years earlier and again developed CCC in the contralateral ovary, which was believed to be either de novo or a recurrence. According to previous reports, cases of recurrence of CCC usually occur within 1 year of the primary tumor [1, 2], and there are no reports of recurrence or de novo development after this much-extended period. Our report highlights the need for long-term monitoring of patients who undergo FSS. In our case, since clear cell adenofibroma was a component of the second CCC, we considered the possibility of de novo development. Our case report discusses this point and presents a review of similar cases presented in previous literature.

Since 2010, studies [3–5] have found that FSS may be recommended for Stage IA CCC patients if suitable staging and postoperative chemotherapy are performed, and a consensus regarding this is gradually taking shape [6, 7]. In Japan, the number of patients with epithelial ovarian cancer (EOC) has been increasing in recent years, and in 2012, 6.8% of patients with this condition had developed it before they reached the age of 40 years [8]. A consequence of the standard treatment for CCC is infertility, but since women today are marrying and having children at a later age, an increasing number of patients are requesting treatment that spares their fertility. Hence, the number of Stage IA CCC patients who undergo FSS is likely to increase.

## Case report

Eleven years ago, when our patient was a 33-year-old unmarried nulligravida, she developed CCC of the ovary for the first time. At that time, her menstrual cycle was regular and 30 days long, with dysmenorrhea, manifesting as lower abdominal and lumbar pain, persisting for 5–7 days. There was no family or medical history of note. She had visited a local clinic with the chief complaint of lumbar pain, where a right ovarian tumor 8 cm in size that included solid internal components, was identified, and she was referred to the University of Tsukuba Hospital. Transvaginal ultrasound had revealed a right adnexal mass with solid components, 68 mm × 53 mm in size, and an intramural uterine fibroid 23 mm × 27 mm, with no hypertrophy of the endometrium, which measured 2.1 mm. Blood counts and blood biochemistry test results revealed no abnormalities. Her serum CA19-9 level was 45.9 U/ml, CA125 was 22 U/ml, and CEA level was 1.3 ng/ml. Abdominal computed tomography (CT) and magnetic resonance imaging (MRI) revealed an 80-mm unilocular cystic mass in the right ovary with a papillary protrusion of longest diameter 45 mm. The left ovary was not enlarged, and there was no obvious peritoneal dissemination or enlarged lymph nodes. Surgery, comprising right adnexectomy, left ovarian biopsy, partial omentectomy and uterine fibroid removal, was performed. The right ovary was enlarged to 8 cm in size, and it was removed without intraoperative rupture and with its capsule intact. The left ovary was not enlarged, but was seen to have a small endometrial cyst, which was resected. The absence of peritoneal dissemination and enlarged lymph nodes was confirmed during surgery. The pathological diagnosis was CCC localized to the right ovary and endometriosis was observed in the left ovary, with ascites cytology Class III. The cytology was composed of clusters that include atypical cells having somewhat nuclear enlargement, and it makes a diagnosis difficult to distinguish mesothelial cells from malignant cells. The patient expressed a strong desire to preserve her fertility. Hence, pelvic and para-aortic lymph node dissections were performed via staged laparotomy, which revealed no metastases in any of the 91 pelvic or para-aortic lymph nodes that were removed, leading to a diagnosis of Stage IA CCC. Four courses of postoperative combination paclitaxel (175 mg/m^2^) and carboplatin (AUC6) chemotherapy (TC therapy) were administered to complete the initial treatment.

**Fig. 1:**
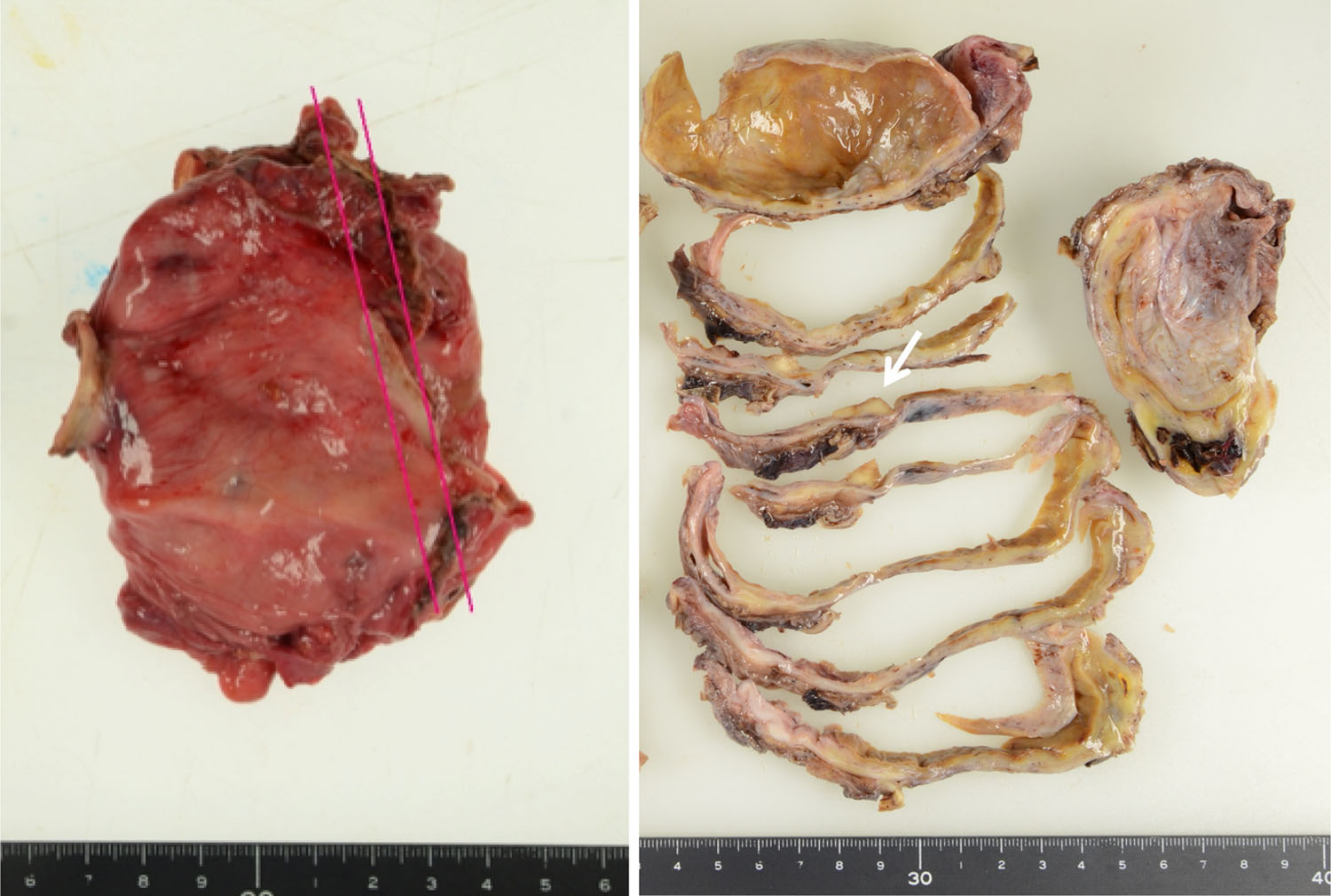
The resected ovarian tumor enlarged to 95 mm in size. The internal lumen of the tumor contained several milky-white mural nodules (*right arrow*) extending around 15 mm into the cavity

**Fig. 2:**
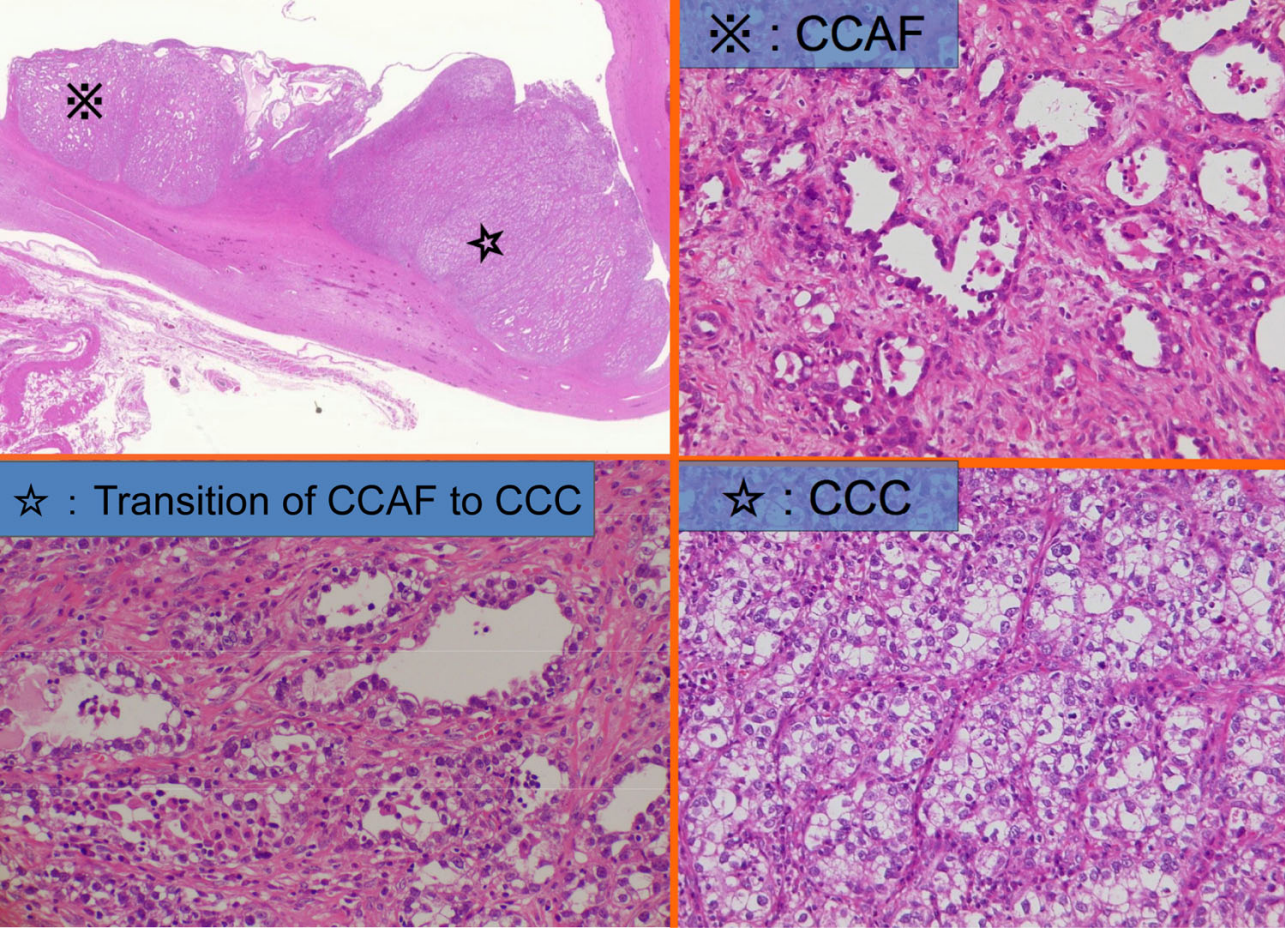
Clear cell adenofibroma (CCAF), transition of CCAF to clear cell carcinoma (CCC) and CCC components in the left ovarian tumor at the patient’s second treatment (HE ×200)

Nine years after the initial therapy, MRI during regular 6-monthly monitoring revealed the appearance of a 95-mm polycystic mass with a mural nodule of longest diameter 53 mm in the left adnexal region. Those findings were never seen at the previous MRI. At this time, her serum CA19-9 level was 12.9 U/ml, CA125 was 18.3 U/ml, and CEA level was 0.5 ng/ml, all of which were within normal limits. Disease recurrence in the contralateral ovary was diagnosed, and total abdominal hysterectomy, left adnexectomy and omental biopsy were performed. At the time of surgery, the left ovary was enlarged to 95 mm in size, and it was removed intact without intraoperative rupture of its capsule. The internal lumen of the tumor contained several milky-white mural nodules extending around 15 mm into the cavity (Fig. 1). The histological diagnosis was CCC, but in addition to the possibility of recurrence, it was also considered that the tumor might have developed de novo, and the fact that clear cell adenofibroma (CCAF) was also present (Fig. 2) suggested that this might have provided the genesis for its development. In addition, there were no endometriotic lesions in the non-solid cyst wall, and it only consists of fibrous membrane. The tumor was localized to the left ovary, and since ascites cytology was negative, it was diagnosed as Stage IA disease. The treatment was completed with four courses of postoperative TC therapy.

Two years after the completion of treatment, the patient is continuing outpatient monitoring with no sign of recurrence to date.

## Discussion

In an analysis of 211 EOC Stage I patients who underwent FSS in Japan, none of the 15 patients with stage IA CCC experienced recurrence, indicating that FSS is possibly a feasible treatment option for these patients [3]; further, the general opinion is that with careful patient selection, FSS is a good treatment option for CCC patients [9]. In the present case, 9 years after FSS, CCC developed in the contralateral ovary. In all five previously reported confirmed cases of recurrence following FSS for Stage IA CCC, [1, 2, 5, 10] recurrence occurred within 1 year, and four of the five lesions were present outside the contralateral ovary (Table 1). The recurrence in the present case was extremely delayed, suggesting the possibility of de novo development from endometriosis of the contralateral ovary at the time of initial surgery. A route of carcinogenesis that does not involve endometriosis has also been suggested, in which CCAF, a benign tumor, develops into CCC via borderline malignant CCAF (atypical cells in the epithelial component of CCAF) [11]. In our case, CCAF was found in specimens of the ovarian tumor removed during left adnexectomy at the time of the second tumor, and it is possible that this was the site that gave rise to de novo carcinoma. The median time to recurrence at a site other than the contralateral ovary is reportedly 14 months (range 1–73 months), whereas for patients with recurrence only in the contralateral ovary, this time is reportedly longer, at 43 months (range 2–172 months) [12], suggesting that the latter group may have included some patients with de novo carcinoma, as in the present case.

**Table 1:** Time to recurrence of patients with recurrence in stage IA clear cell carcinoma

Author	Year	*n*	TTR (months)	Site of recurrence	Status
Morice [1]	2005	1	6	Extra-ovary	AWD
Park [2]	2008	2	11	Extra-ovary	DOD
			9	Extra-ovary	AWD
Fruscio [5]	2013	1	8	Extra-ovary	NED
Ditto [10]	2014	1	7	Residual ovary	NED

*TTR* time to recurrence, *NED* no evidence of disease, *AWD* alive with disease, *DOD* died of disease

Mortality among patients with recurrence at a site other than the contralateral ovary after FSS for ovarian carcinoma has been reported to be 62 or 82%, compared with only 13 or 19% for those who develop recurrence only in the contralateral ovary, which is a considerably better figure [12, 13]. We added data to those reports from other studies which were not sited in them that clearly stated the association between the site of recurrence and prognosis, and we have summarized this data in Table 2, including the proportion of patients surviving with no evidence of disease (NED) after salvage therapy for each site of recurrence. Since the figures are simple calculations from studies that differ in aspects such as monitoring periods, they cannot be described as highly reliable, but they do show a better prognosis for patients with recurrence in the contralateral ovary alone, 75.9% of whom exhibited NED, compared with a NED rate of only 16.7% in those with recurrence at a site other than the contralateral ovary.

**Table 2:** Survival rate of NED in patients receiving FSS with stage I EOC

References	Recurrence site					
	Contralateral ovary alone			Other sites with/without ovary		
	NED	AWD	DOD	NED	AWD	DOD
Zanetta [14]	1	0	0	0	1	3
Jobo [15]	0	0	2	0	0	1
Schilder [16]	3	0	0	0	0	2
Colombo [17]	0	0	0	0	0	1
Morice [1]	2	0	2	2	2	1
Borgfeldt [18]	0	0	0	0	0	1
Park [2]	1	0	0	1	3	4
Schlaerth [19]	0	0	3	0	0	0
Anchezar [20]	0	0	0	1	0	1
Kajiyama [21]	1	0	1	0	0	6
Satoh [3]	5	0	0	3	5	5
Cheng [22]	0	0	0	1	0	0
Kashima [23]	1	0	0	0	0	4
Fruscio [5]	6	1	0	3	0	11
Ditto [10]	2	0	0	0	0	0
Survival rate of NED	71.0% (22/31)			17.7 (11/62)		

*NED* no evidence of disease, *FSS* fertility-sparing surgery, *EOC* epithelial ovarian cancer, *AWD* alive with disease, *DOD* died of disease

The present patient was enrolled in a retrospective study [3] that found that not one of the 15 patients with Stage IA CCC experienced recurrence, and her lesion in the left ovary appeared after that paper was published. According to previous reports, the patients with Stage IA CCC who underwent FSS developed recurrence in about 1 year [1, 2]; hence, ours is the first report of appearance of a lesion 9 years after FSS. Ultimately, whether it represented recurrence or de novo development remains unknown, but in either case, the fact that salvage therapy alone after the appearance of a lesion in the contralateral ovary offers the prospect of a good prognosis indicates that patients who have undergone FSS must be monitored carefully over the long term, so that salvage therapy can be initiated while the lesion is still restricted to the contralateral ovary.
